# Autologous transplant vs. CAR-T therapy in patients with DLBCL treated while in complete remission

**DOI:** 10.1038/s41408-024-01084-w

**Published:** 2024-07-08

**Authors:** Mazyar Shadman, Kwang W. Ahn, Manmeet Kaur, Lazaros Lekakis, Amer Beitinjaneh, Madiha Iqbal, Nausheen Ahmed, Brian Hill, Nasheed M. Hossain, Peter Riedell, Ajay K. Gopal, Natalie Grover, Matthew Frigault, Jonathan Brammer, Nilanjan Ghosh, Reid Merryman, Aleksandr Lazaryan, Ron Ram, Mark Hertzberg, Bipin Savani, Farrukh Awan, Farhad Khimani, Sairah Ahmed, Vaishalee P. Kenkre, Matthew Ulrickson, Nirav Shah, Mohamed A. Kharfan-Dabaja, Alex Herrera, Craig Sauter, Mehdi Hamadani

**Affiliations:** 1 Clinical Research Division, Fred Hutchinson Cancer Center, Seattle, WA USA; 2 Division of Hematology and Medical Oncology, University of Washington, Seattle, WA USA; 3 Center for International Blood and Marrow Transplant Research, Medical College of Wisconsin, Milwaukee, WI USA; 4 Division of Biostatistics, Institute for Health and Equity, Medical College of Wisconsin, Milwaukee, WI USA; 5 Division of Transplantation and Cellular Therapy, University of Miami Hospital and Clinics, Sylvester Comprehensive Cancer Center, Miami, FL USA; 6 Division of Hematology and Oncology, Mayo Clinic, Jacksonville, FL USA; 7 Division of Hematologic Malignancies & Cellular Therapeutics, University of Kansas Cancer Center, Westwood, KS USA; 8 Department of Hematology and Medical Oncology, Taussig Cancer Institute, Cleveland Clinic, Cleveland, OH USA; 9 Division of Hematology-Oncology, Hospital of the University of Pennsylvania, Philadelphia, PA USA; 10 Department of Medicine, Section of Hematology and Oncology, University of Chicago, Chicago, IL USA; 11 Lineberger Comprehensive Cancer Center, Department of Medicine, Hematology Oncology, University of North Carolina School of Medicine, Chapel Hill, NC USA; 12 Hematopoietic Cell Transplant and Cell Therapy Program, Massachusetts General Hospital, Boston, MA USA; 13 Division of Hematology, Department of Internal Medicine, James Comprehensive Cancer Center, The Ohio State University, Columbus, OH USA; 14 Levine Cancer Institute, Atrium Health, Charlotte, NC USA; 15 Department of Medical Oncology, Dana-Farber Cancer Institute, Boston, MA USA; 16 Department of Blood and Marrow Transplant and Cellular Immunotherapy, Moffitt Cancer Center and Research Institute, Tampa, FL USA; 17 Bone Marrow Transplantation Unit, Tel Aviv Sourasky Medical Center, Tel Aviv, Israel; 18 Sackler Faculty of Medicine, Tel Aviv University, Tel Aviv, Israel; 19 Prince of Wales Hospital, Sydney, NSW Australia; 20 Long Term Transplant Clinic, Vanderbilt University Medical Center, Nashville, TN USA; 21 Division of Hematology and Oncology, UT Southwestern, Dallas, TX USA; 22 Department of Lymphoma/Myeloma, The University of Texas MD Anderson Cancer Center, Houston, TX USA; 23 Department of Stem Cell Transplantation & Cellular Therapy, The University of Texas MD Anderson Cancer Center, Houston, TX USA; 24 Division of Hematology, Oncology, Palliative Care, Department of Medicine, University of Wisconsin, Madison, WI USA; 25 Banner MD Anderson Cancer Center, Gilbert, AZ USA; 26 BMT & Cellular Therapy Program, Department of Medicine, Medical College of Wisconsin, Milwaukee, WI USA; 27 Division of Lymphoma, Department of Hematology and Hematopoietic Cell Transplantation, City of Hope, Duarte, CA USA

**Keywords:** Cancer immunotherapy, Combination drug therapy

## Abstract

In patients with relapsed DLBCL in complete remission (CR), autologous hematopoietic cell transplantation (auto-HCT) and CAR-T therapy are both effective, but it is unknown which modality provides superior outcomes. We compared the efficacy of auto-HCT vs. CAR-T in patients with DLBCL in a CR. A retrospective observational study comparing auto-HCT (2015–2021) vs. CAR-T (2018–2021) using the Center for International Blood & Marrow Transplant Research registry. Median follow-up was 49.7 months for the auto-HCT and 24.7 months for the CAR-T cohort. Patients ages 18 and 75 with a diagnosis of DLBCL were included if they received auto-HCT (*n* = 281) or commercial CAR-T (*n* = 79) while in a CR. Patients undergoing auto-HCT with only one prior therapy line and CAR-T patients with a previous history of auto-HCT treatment were excluded. Endpoints included Progression-free survival (PFS), relapse rate, non-relapse mortality (NRM) and overall survival (OS). In univariate analysis, treatment with auto-HCT was associated with a higher rate of 2-year PFS (66.2% vs. 47.8%; *p* < 0.001), a lower 2-year cumulative incidence of relapse (27.8% vs. 48% ; *p* < 0.001), and a superior 2-year OS (78.9% vs. 65.6%; *p* = 0.037). In patients with early (within 12 months) treatment failure, auto-HCT was associated with a superior 2-year PFS (70.9% vs. 48.3% ; *p* < 0.001), lower 2-year cumulative incidence of relapse (22.8% vs. 45.9% ; *p* < 0.001) and trend for higher 2-year OS (82.4% vs. 66.1% ; *p* = 0.076). In the multivariable analysis, treatment with auto-HCT was associated with a superior PFS (hazard ratio 1.83; *p* = 0.0011) and lower incidence of relapse (hazard ratio 2.18; *p* < 0.0001) compared to CAR-T. In patients with relapsed LBCL who achieve a CR, treatment with auto-HCT is associated with improved clinical outcomes compared to CAR-T. These data support the consideration of auto-HCT in select patients with LBCL achieving a CR in the relapsed setting.

## Introduction

Chimeric antigen receptor T-cell (CAR-T) therapy is one of the standard of care (SOC) options for the treatment of patients with relapsed diffuse large B-cell lymphoma (DLBCL) after at least two lines of therapy, and recently, axicabtagene ciloleucel (axi-cel) and lisocabtagene maraleucel (liso-cel) received the approval by the FDA to be used in second-line for patients with refractory disease or if relapsing within 12 months of first-line treatment [1–6]. The latter approval was based on randomized trials that showed the superiority of axi-cel and liso-cel compared to SOC consisting of salvage chemotherapy followed by high-dose chemotherapy and autologous hematopoietic cell transplant (auto-HCT) [7]. Auto-HCT remains SOC for patients with late relapse who meet the transplant eligibility criteria [1, 8–11].

In practice, patients who are considered CAR-T candidates commonly receive interim or bridging chemotherapy before having access to CAR-T [12]. Occasionally, these patients achieve a response to chemotherapy, sometimes in the form of a complete remission (CR). With chemosensitive disease, auto-HCT is potentially an option for such patients. On the other hand, recent reports indicate durable remissions in patients who receive CAR-T therapy while in a CR [13–15]. These findings have raised questions about the comparative performance of auto-HCT vs. CAR-T therapy in CR patients who experience early or late relapse. Data from randomized trials do not directly inform the clinical decisions in this setting. We have previously reported superior outcomes in patients who received auto-HCT compared to those who received CAR-T therapy in partial remission (PR) [16, 17]. Herein, to further investigate the comparative effectiveness of auto-HCT versus CAR-T therapy in patients with chemosensitive relapsed DLBCL, we used the Center for International Blood and Marrow Transplant Research (CIBMTR) database to compare the outcome of patients with LBCL who received auto-HCT or CAR-T after attaining a CR.

## Methods

### Data source

For details of the data source, please refer to the supplemental material available on the *Blood Cancer Journal* Web site. This study is approved by the Medical College of Wisconsin institutional review board.

### Study design and population

Patients between ages 18 and 75 with a diagnosis of large B-cell lymphoma (LBCL) including DLBCL, primary mediastinal lymphoma, and high-grade B-cell lymphoma with MYC and/or BCL2/BCL6 rearrangement were included if they received auto-HCT between 2015-2021 or commercial CAR-T therapy with tisagenlecleucel (tisa-cel), axi-cel, or liso-cel between 2018-2021 while in a CR by CT or PET scan and per the 2014 Lugano definition [18]. Patients undergoing auto-HCT with only one prior therapy line in CR1 were excluded. We also excluded patients in the CAR-T group with a previous history of auto-HCT treatment.

### Exposure and outcome

The primary endpoints of the study were progression-free survival (PFS) and overall survival (OS). PFS was defined as the time from either auto-HCT or CAR-T to relapse, progression or death from any cause. OS was defined as the time from treatment to death from any cause. Non-relapse mortality (NRM) was defined as death without evidence of prior lymphoma progression/relapse; relapse was considered a competing risk. Progression/relapse was defined as relapsed lymphoma after CAR-T or autoHCT; NRM was considered a competing risk. For CAR-T patients, the cumulative incidence of cytokine release syndrome (CRS) and immune effector cell–associated neurotoxicity syndrome (ICANS) were calculated. CR status before auto-HCT or CAR-T was defined per Lugano working group classification and determined by local radiologic assessments. Grading of CRS and ICANS was done according to the American Society of Transplant and Cellular Therapy grading criteria [18, 19]. Subgroup analyses were performed for patients with early treatment failure in 12 months, defined by having primary refractory disease to first-line chemotherapy or relapse within 12 months of starting chemotherapy.

### Statistical analysis

The baseline characteristics were compared between the auto-HCT and CAR-T groups using the Kruskal-Wallis test for continuous variables and the Pearson X^2^ test for categorical variables after ignoring the missing data. The PFS and OS of the auto-HCT and CAR-T groups were compared using the Kaplan-Meier estimator and the log-rank tests. The NRM and relapse/progression rates in the two cohorts were compared using the cumulative incidence function with Gray’s test to account for competing events. The Cox proportional hazard model was used to compare PFS and OS, and the proportional cause-specific hazards model was used to compare the NRM and relapse/progression in two groups. The variables included in the regression model were age (continuous and by decade), sex, race (Caucasian vs. others), Karnofsky performance status (KPS) (≥90 vs. <90 vs. missing), refractoriness to first-line treatment, early failure (primary refractory or relapse in < 1 year of first-line treatment), number of lines of prior therapies. The forward stepwise selection was used to identify significant variables at a significance level of 0.05. The interaction between auto-HCT/CAR-T groups and the other significant variables was examined. The proportional hazards assumption was examined by testing covariates’ time-varying effects. All statistical analyses were performed using SAS version 9.4 (SAS Institute, Cary, NC) and R version 4.0.4 (R Foundation for Statistical Computing, Vienna, Austria).

## Results

### Baseline characteristics

We identified 360 patients with LBCL who received auto-HCT (n = 281) or CAR-T (*n* = 79) while in a CR, and baseline characteristics are shown in Table 1. In the auto-HCT group, a smaller proportion of patients had a history of early (12 months) treatment failure after first-line therapy (58% vs. 72.2%; *p* = 0.02), and the number of prior treatment lines was fewer (median 2 vs. 3; *p* < 0.01) compared to the CAR-T group. More patients from the auto-HCT group had a KPS of 90% or more compared to the CAR-T cohort (57.3% vs. 39.2% ; *p* < 0.1). The two groups were balanced for patients’ age (median 59 vs. 64 years; *p* = 0.14), female sex (36.7% vs. 40.5%; *p* = 0.53) and proportion of patients with primary refractory disease (20% vs. 29%; *p* = 0.22). No patients received CAR-T therapy as second-line treatment. CR was determined by PET or PET/CT in 99.6% of auto-HCT patients and 89.9% of CAR-T patients (*p* < 0.01). Two patients from the CAR-T group received allogeneic transplant after CAR-T therapy and no patient had auto-HCT. Median follow-up for survivors was 49.7 months for the auto-HCT group and 24.7 months for the CAR-T group.

**Table 1:** Baseline characteristics of DLBCL patients who received auto-HCT or CAR-T while in a CR.

Characteristic	Auto-HCT (*n* = 281)	CAR-T (*n* = 79)	*P* Value
Age			0.14^a^
Median (min–max)	59.4 (18.2–75.6)	64.1 (20.1–76.0)	
>65 - no. (%)	92 (32.7)	35 (44.3)	
Female Sex - no. (%)	103 (36.7)	32 (40.5)	0.53^a^
Race - no. (%)			<0.01^a^
White	192 (68.3)	61 (77.2)	
Black or African American	41 (14.6)	2 (2.5)	
Asian	36 (12.8)	1 (1.3)	
Others*	12 (4.3)	15 (19.0)	
Karnofsky score prior 90–100 - no. (%)	161 (57.3)	31 (39.2)	<0.01^a^
Not reported	6 (2.1)	20 (25.3)	
High-grade B-cell lymphoma, with MYC and BCL2 and/or BCL6 rearrangements - no. (%)			0.03^a^
Yes	31 (27.0)	11 (13.9)	
No	84 (73.0)	68 (86.1)	
Not collected before 2018	166 (0.0)	0 (0.0)	
Advanced Stage at diagnosis - no. (%)	186 (75.3)	37 (64.9)	0.11^a^
Elevated LDH before auto-HCT or CAR-T - no. (%)	86 (30.6)	29 (36.7)	0.04^a^
Not reported	150 (53.4)	30 (38.0)	
Refractory to first line therapy - no. (%)			0.22^a^
No	180 (64.1)	45 (57.0)	
Yes	56 (19.9)	23 (29.1)	
Not assessed/not reported	45 (16.0)	11 (13.9)	
Early therapy failure in 12 months - no. (%)	163 (58.0)	57 (72.2)	0.02^a^
Total lines of therapies - median (min-max)	2.0 (2.0–8.0)	3.0 (2.0–8.0)	<0.01^b^
PET or PET/CT performed prior to HCT/CAR-T to assess disease status - no. (%)	280 (99.6)	71 (89.9)	<0.01^a^
Product - no. (%)			N/A
tisagenlecleucel	0 (0.0)	42 (53.2)	
axicabtagene ciloleucel	0 (0.0)	36 (45.6)	
lisocabtagene maraleucel	0 (0.0)	1 (1.3)	
Year of auto-HCT/CAR-T - no. (%)			<0.01^a^
2018 and after	114 (40.5)	79 (100)	
Follow-up among survivors, months - median (range)	49.7 (3.0–95.4)	24.7 (3.3–49.4)	

Hypothesis testing: ^a^Pearson chi-square test ^b^Kruskal-Wallis test.

*HCT cohort: American Indian or Alaska Native (*n* = 6), multiple race (*n* = 2), and not reported (*n* = 4). CAR-T cohort: Multiple race (*n* = 6), and not reported (*n* = 9).

### Univariable analysis

**Fig. 1:**
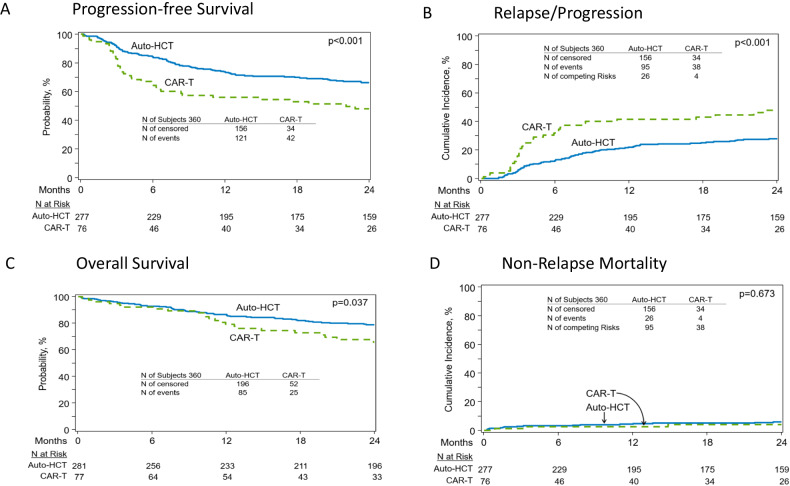
Auto-HCT vs CAR-T in patients with DLBCL in CR. **A** progression-free survival. **B** Cumulativeincidence of relapse. **C** Overall survival. **D**. Non-relapse Mortality.

Patients who received auto-HCT while in a CR had a superior PFS [2-year PFS 66.2% (95% CI: 60.4–71.8) vs. 47.8% (95% CI: 36.4–59.4); *p* < 0.001], lower cumulative incidence of relapse/progression [2-year release/progression rate 27.8% (95% CI: 22.6–33.4) vs. 48% (95% CI: 36.4–59.7); *p* < 0.001] and a better 2-year OS [78.9% (95% CI: 73.9–83.6) vs. 65.6% (95% CI: 53.6–76.6); *p* = 0.037] compared to the CAR-T group. (Fig. 1A–C; Table 2) There was no statistical difference in the cumulative incidence of NRM between the two groups at 1 year [4.4% (95% CI: 2.3–7.1) vs. 2.6% (95% CI: 0.2–7.4)] or 2 years [(5.9% (95% CI: 3.4–9.1) vs. 4.1%(95% CI: 0.8–10)] (*p* = 0.673). (Fig. 1B; Table 2)

**Table 2:** Univariable analysis of outcomes in patients treated with auto-HCT or CAR-T while in a CR.

Outcomes					
	Auto-HCT		CAR-T		*P*-value
	*N*	Prob (95%CI)	N	Prob (95%CI)	
Non-relapse mortality	277		76		0.673
1-year		4.4% (2.3-7.1)		2.6% (0.2-7.4)	
2-year		5.9% (3.4-9.1)		4.1% (0.8-10)	
Relapse/progression	277		76		<0.001
1-year		21.9% (17.2-27)		41.6% (30.6-53.1)	
2-year		27.8% (22.6-33.4)		48% (36.4-59.7)	
Progression-free survival	277		76		<0.001
1-year		73.7% (68.3-78.8)		55.7% (44.4-66.8)	
2-year		66.2% (60.4-71.8)		47.8% (36.4-59.4)	
Overall survival	281		77		0.037
1-year		86.7% (82.4-90.4)		79.1% (69-87.7)	
2-year		78.9% (73.9-83.6)		65.6% (53.6-76.6)	

### Subgroup analyses

In an analysis that was limited to patients with a refractory disease after first-line treatment or those who experienced relapse within 12 months and received auto-HCT (*n* = 181) or CAR-T (*n* = 63) in a CR, the 2-year PFS was superior in the auto-HCT [68.2% (95% CI: 61.1–74.9) vs. 48.4% (95% CI: 35.7–61.3); *p* = 0.001] and the 2-year cumulative incidence of progression/relapse was lower in auto-HCT [25% (95% CI: 18.8–31.7) vs. 46.3% (95% CI: 33.5–59.3); *p* < 0.001]. Although not statistically significant, the 2-year OS was numerically higher in the auto-HCT group compared to the CAR-T group [79.6% (95% CI: 73.3–85.2) vs. 66.3% (95% CI: 53–78.4); *p* = 0.131). (Table 3)

**Table 3:** Univariable analysis of outcomes in patients who relapsed within 12 months of first-line treatment and were treated with auto-HCT or CAR-T while in a CR.

Outcomes					
	Auto-HCT		CAR-T		*P*-value
	*N*	Prob (95%CI)	*N*	Prob (95%CI)	
Non-relapse mortality	179		60		0.771
1-year		5.6% (2.7–9.5)		3.3% (0.3–9.4)	
2-year		6.8% (3.6–11)		5.3% (1–12.7)	
Relapse/progression	179		60		<0.001
1-year		21.9% (16.1–28.3)		46.3% (33.5–59.3)	
2-year		25% (18.8–31.7)		46.3% (33.5–59.3)	
Progression-free survival	179		60		0.001
1-year		72.5% (65.7–78.8)		50.4% (37.6–63.1)	
2-year		68.2% (61.1–74.9)		48.4% (35.7-61.3)	
Overall survival	181		61		0.131
1-year		84.4% (78.7–89.3)		78.8% (67.2–88.4)	
2-year		79.6% (73.3–85.2)		66.3% (53–78.4)	

### Multivariable analysis

In the multivariable regression analysis, treatment with auto-HCT was associated with a lower risk of relapse/progression [Hazard ratio (HR) 2.18 (95% CI: 1.48-–3.2); *p* < 0.0001] and a superior PFS [HR 1.83 (95% CI: 1.27–2.63); *p* = 0.0011] compared to CAR-T. There was no significant difference between auto-HCT and CAR-T groups for NRM [HR 0.59 (95% CI: 0.19–1.83); *p* = 0.36] and OS [HR 1.44 (95 CI: 0.91–2.28); *p* = 0.12]. Table 4. There was no significant difference between the outcomes of tisa-cel (reference group) and axi-cel in term of NRM [HR = 1.08 (95% CI: 0.15–7.12); *p* = 0.94], relapse [HR 0.53 (95% CI: 0.27–1.06); *p* = 0.07], PFS [HR 0.57 (95% CI: 0.30–1.09); *p* = 0.09] and OS [HR 0.51 (95% CI: 0.21–1.230; *p* = 0.13).

**Table 4:** Multivariable regression analysis of outcomes in patients treated with auto-HCT or CAR-T while in a CR.

Outcomes					
	*N*	HR	Lower limit 95% CI	Higher limit 95% CI	*P*-value
**Non-relapse mortality**					
Age, year					<0.0001
18–40	33	1			
41–65	194	1.11	0.14	9.06	
>65	127	6.41	0.85	48.24	
Refractoriness to first line					0.0306
No	220	1			
Yes	79	2.97	1.30	6.76	
Missing	55	1.99	0.70	5.63	
Main effect					0.3632
Auto-HCT	277	1			
CAR-T	77	0.59	0.19	1.83	
**Relapse/progression**					
Main effect					<.0001
Auto-HCT	277	1			
CAR-T	77	2.18	1.48	3.20	
**Progression-free survival**					
Age, year					<0.0001
18–40		1			
41–65		1.30	0.67	2.52	
>65		1.99	1.03	3.86	
Main effect					0.0011
Auto-HCT	277	1			
CAR-T	77	1.83	1.27	2.63	
**Overall survival**					
Age, year					0.0002
18–40	34	1			
41–65	197	1.19	0.51	2.79	
>65	127	2.56	1.10	5.95	
Main effect					0.1234
Auto-HCT	281	1			
CAR-T	77	1.44	0.91	2.28	

### Toxicity and causes of death

In the CAR-T group, 48 patients (68.4%) developed CRS (grade 3-4: 2; 2.6%), and 25 (31.6%) developed ICANS (grade 3-4: 5; 6.3%). (Supplement, Table 1) During the follow-up, 85 patients (30.2%) of auto-HCT patients and 25 patients (31.6%) of CAR-T patients died. The most common causes of death in both groups were first, related to disease progression [auto-HCT: 51 (60%) and CAR-T: 17 (68%)] and second, to infections [auto-HCT: 13 (15.3%) and CAR-T: 2 (8%)]. Other common causes of death included cardiac failure (*n* = 3, 3.5%) and second primary malignancies (*n* = 3, 3.5%) in the auto-HCT group and ICANS (*n* = 1, 4%) and second primary malignancies (*n* = 3, 3.5%) in the CAR-T group. COVID-19 was responsible for 1.2% and 4% of deaths in the auto-HCT and CAR-T groups, respectively. (Supplement, Table 2)

## Discussion

In this retrospective comparative study using the CIBMTR registry, we show that the outcome of patients with relapsed LBCL who receive an auto-HCT while in a CR is superior to those who receive CAR-T therapy, both in CR. Auto-HCT is associated with a lower incidence of relapse/progression, a longer PFS, and a superior OS. These associations were observed in the overall study population and also when the analysis was confined to patients with primary refractory disease or those with relapsed disease within 12 months. Auto-HCT remained independently associated with a lower relapse rate and an improved PFS in the multivariable analysis.

CAR-T therapy (tisa-cel, axi-cel, and liso-cel) is considered the SOC for patients with relapsed LBCL in patients after two prior lines of treatment [2–4]. Axi-cel and liso-cel are also approved by the FDA for sconed-line in patients with primary refractory disease based on ZUMA-7 and TRANSFORM studies, respectively [7, 9]. These studies compared CAR-T with the SOC consisting of second-line chemotherapy followed by auto-HCT in patients with PR or CR. In both studies, more than half of the patients on the SOC arms were considered to have a “failure event” because of chemorefractory disease per study protocols before receiving auto-HCT. Therefore, the two trials were not poised to compare the efficacy of auto-HCT vs. CAR-T in patients who were eligible for auto-HCT defined by having a chemosensitive disease in the form of a PR or a CR. Our findings are not contradictory to the results of the mentioned randomized studies as it address a different clinical question relevant to patients with proven chemosensitive disease. These findings are consistent with our previous findings, indicating higher efficacy (lower relapse rate and a longer PFS) of auto-HCT compared to CAR-T in patients in partial remission [16]. Comparing patients in CR in the current study eliminates the potential confounding associated with the heterogenicity of PRs in the prior analysis which was a valid criticism of that study [20]. Overall, both studies argue for the continued role of auto-HCT in patients with LBCL with chemosensitive disease in the relapsed setting with utilizing CAR-T in the post-auto-HCT setting if needed.

In clinical practice, timing and access to CAR-T differ from the clinical trials. Based on one report, 40% of patients who intended to receive CAR-T did not receive it for a variety of reasons, with disease progression as the most common cause [21]. Therefore, administration of interim chemotherapy, defined as treatments that are administered after a decision for CAR-T was made until CAR-T infusion, is not an uncommon scenario and may lead to CRs in some patients. Different groups have reported the feasibility, safety, and high activity of CAR-T therapy in patients in a CR. First, Investigators from the JULIET trial reported that seven patients treated with tisa-cel while in a CR remained in a CR three months after CAR-T infusion, and 5 of 7 remained disease-free after one year [14]. Jallouk and colleagues later reported on 13 patients who received axi-cel while in CR by PET at the MD Anderson Cancer Center. Comparing these patients with a matched cohort of LBCL patients who received axi-cel with PET avid disease, the authors did not find a difference in the PFS and OS between the two groups with a median follow-up of 26 months [13]. In another report from 8 academic US centers, Wudhikarn et al. reported on 33 patients who received axi-cel or tisa-cel while in a CR by PET and had a 1-year PFS of 60% [15]. Two of these studies described CAR-T expansion and did not report an inferior CAR-T expansion/amplification profile in these small series. To our knowledge, our study is the first analysis comparing the long-term outcomes of patients receiving auto-HCT vs. CAR-T while in a CR.

Our study has several limitations. As a retrospective analysis, investigators were limited in eliminating potential confounders that may influence the association between the treatment modality and outcomes. Ideally, a randomized clinical trial comparing auto-HCT with CAR-T for patients in a CR would inform the practice in this setting. In the absence of a randomized trial, an analysis using the CIBMTR registry provides a high-quality, real-world analysis. Also, more than half of the patients in the CAR-T cohort received tisa-cel, which may have lower efficacy than other approved CAR-T products and can underestimate the efficacy of CAR-T in this analysis [22, 23]. In the CAR-T cohort, there was no statistical difference between the efficacy of axi-cel vs. tisa-cel but a repeat analysis in future by including more patients treated with axi-cel or liso-cel may address this issue in the future.

These results may inform, or validate, clinical practices in select settings. In patients experiencing late relapses (after 12 months) that are transplant-eligible from a comorbidity standpoint, auto-HCT should remain SOC as there is currently no evidence to indicate better outcomes with CAR-T. In patients with primary refractory disease or early relapse, CAR-T should be the goal of therapy, improving access to CAR-T should remain a priority, and strategies to minimize the time to CAR-T therapy should be implemented [24]. As efforts to reach those goals continue, in the subset of patients who achieve a CR with interim treatment, a discussion about the possibility of utilizing auto-HCT seems reasonable and can provide a curative option for some patients while keeping CAR-T as a backup treatment plan in case of auto-HCT failure.

### Supplementary information

Supplementary Material

## Data Availability

CIBMTR data related to this work will be publicly available online on the CIBMTR website after publication.
