# Supplementary material for: Autologous transplant vs. CAR-T therapy in patients with DLBCL treated while in complete remission
Source: Blood Cancer J. 2024 Jul 8;14(1):108. doi: 10.1038/s41408-024-01084-w (PMC11231252; doi:10.1038/s41408-024-01084-w)
Supplement: Supplementary file 1 — Supplementary Material [file 41408_2024_1084_MOESM1_ESM.docx]

**Supplementary Material**

**Data source**

The CIBMTR is a working group comprised of over 380 transplantation centers worldwide that provide data regarding HCT and cellular therapies to a statistical center at the Medical College of Wisconsin (MCW). On-site audits ensure compliance of participating transplant centers in reporting all transplantations consecutively. Additionally, quality of data is further augmented through computerized affirmation of discrepancies, physicians’ review of submitted data, and on-site audits of participating centers. Observational studies are conducted by the CIBMTR in compliance with all pertinent federal regulations with regards to protection of human research participants. All patients included in this analysis have provided written consent for research. The Institutional Review Board of MCW have approved this study.

S1. CRS and ICANS in the CAR-T group

| **Characteristic** | **N (%)** |
| --- | --- |
| No. of patients | 79 |
| Maximum CRS grade (during follow-up for this CT) - no. (%) |  |
| No CRS | 25 (31.6) |
| Grade 1 | 31 (39.2) |
| Grade 2 | 19 (24.1) |
| Grade 3 | 1 (1.3) |
| Grade 4 | 1 (1.3) |
| Not reported | 2 (2.5) |
| Time to CRS, days - median (min-max) | 3.0 (1.0-16.0) |
| Maximum neurotoxicity grade (during follow-up for this CT) - no. (%) |  |
| No neurologic impairment | 54 (68.4) |
| Grade 1 | 8 (10.1) |
| Grade 2 | 6 (7.6) |
| Grade 3 | 2 (2.5) |
| Grade 4 | 3 (3.8) |
| Not reported | 6 (7.6) |
| Time to ICANS, days - median (min-max) | 7.0 (2.0-13.0) |

S2. Causes of death

| **Characteristic** | **Auto-HCT** | **CAR-T** |
| --- | --- | --- |
| No. of patients | 281 | 79 |
| Overall survival - no. (%) |  |  |
| No | 196 (69.8) | 52 (65.8) |
| Yes | 85 (30.2) | 25 (31.6) |
| Not reported | 0 (0.0) | 2 (2.5) |
| Primary cause of death - no. (%) |  |  |
| Infection, organism not identified | 4 (4.7) | 1 (4.0) |
| Bacterial infection | 6 (7.1) | 0 (0.0) |
| Fungal infection | 3 (3.5) | 1 (4.0) |
| COVID-19 (SARS-CoV-2) | 1 (1.2) | 1 (4.0) |
| Recurrence/persistence/progression of primary disease for which the HCT/CT was given | 51 (60.0) | 17 (68.0) |
| Cardiac failure | 3 (3.5) | 0 (0.0) |
| Pulmonary failure | 0 (0.0) | 2 (8.0) |
| Central nervous system (CNS) failure | 1 (1.2) | 0 (0.0) |
| Multiple organ failure | 1 (1.2) | 0 (0.0) |
| New malignancy (post-HCT/CT) | 3 (3.5) | 1 (4.0) |
| Intracranial hemorrhage | 1 (1.2) | 0 (0.0) |
| Thromboembolic | 1 (1.2) | 0 (0.0) |
| Prior malignancy (initially diagnosed prior to HCT/CT, other than the malignancy for which the HCT/CT was performed) | 1 (1.2) | 0 (0.0) |
| Neurotoxicity (ICANS) | 0 (0.0) | 1 (4.0) |
| Other cause | 7 (8.2) | 1 (4.0) |
| Not reported | 2 (2.4) | 0 (0.0) |
